# Natural and induced loss of function mutations in *SlMBP21* MADS-box gene led to *jointless-2* phenotype in tomato

**DOI:** 10.1038/s41598-017-04556-1

**Published:** 2017-06-30

**Authors:** Maria Victoria Gomez Roldan, Claire Périlleux, Halima Morin, Samuel Huerga-Fernandez, David Latrasse, Moussa Benhamed, Abdelhafid Bendahmane

**Affiliations:** 1 Institute of Plant Sciences Paris-Saclay (IPS2), CNRS, INRA, University Paris-Sud, University of Evry, University Paris-Diderot, Sorbonne Paris-Cite, University of Paris-Saclay, 91405 Orsay, France; 2 InBioS, PhytoSYSTEMS, Laboratory of Plant Physiology, University of Liège, Sart Tilman Campus Quartier Vallée 1, Chemin de la Vallée 4, B-4000 Liège, Belgium

## Abstract

Abscission is the mechanism by which plants disconnect unfertilized flowers, ripe fruits, senescent or diseased organs from the plant. In tomato, pedicel abscission is an important agronomic factor that controls yield and post-harvest fruit quality. Two non-allelic mutations, *jointless* (*j*) and *jointless-2* (*j-2*), controlling pedicel abscission zone formation have been documented but only *j-2* has been extensively used in breeding. *J* was shown to encode a MADS-box protein. Using a combination of physical mapping and gene expression analysis we identified a positional candidate, *Solyc12g038510*, associated with *j-2* phenotype. Targeted knockout of *Solyc12g038510*, using CRISPR/Cas9 system, validated our hypothesis. *Solyc12g038510* encodes the MADS-box protein SlMBP21. Molecular analysis of *j-2* natural variation revealed two independent loss-of-function mutants. The first results of an insertion of a *Rider* retrotransposable element. The second results of a stop codon mutation that leads to a truncated protein form. To bring new insights into the role of *J* and *J-2* in abscission zone formation, we phenotyped the single and the double mutants and the engineered alleles. We showed that *J* is epistatic to *J-2* and that the branched inflorescences and the leafy sepals observed in accessions harboring *j-2* alleles are likely the consequences of linkage drags.

## Introduction

Abscission is common in plants which thereby separate senescent or damaged organs from their main body, regulate fruit load and release ripe fruits for seed dispersal. Separation is achieved by cell wall degradation between specialised cell layers forming the abscission zone (AZ) of leaves or flower parts. Before the shedding process itself, AZ cells resemble meristematic cells, being small with dense cytoplasm, lacking large vacuoles and any aspect of differentiation. Further development of the AZ proceeds by acquisition of competence to respond to abscission signals, execution of the abscission step *per se* by cell wall loosening and cell expansion, and formation of a protective lignified layer^1, 2^.

If abscission has important functions in plant development and reproduction, its suppression facilitates fruit harvest in crop production. In tomato, floral stems that remain attached to harvested fruits during picking mechanically damage the fruits during transportation, decreasing the fruit quality for fresh-market tomatoes and the pulp quality for processing tomatoes. In mutants that lack the flower AZ, pedicels and calyxes remain attached to the inflorescence axis so that fruits are harvested without any green tissues and can be more easily processed. The jointless pedicel trait has been successfully introgressed in small-fruited processing and fresh-market type’s tomatoes. In contrast, introgression of jointless trait in large-fruited tomatoes was more difficult to develop^3^.

Classical genetics showed that the jointless phenotype is caused, at least, by two independent loci called *jointless* (*j*) and *jointess-2* (*j-2*)^4, 5^. The *j* mutant was first described in a domestic cultivar^6^ and shown to be pleiotropic. In addition to its pedicel AZ phenotype, *j* mutant shows alteration in inflorescence architecture. In *j* mutant, the typical truss is indeed converted into an inflorescence made of leaves and flowers due to the resumption of vegetative meristems in place of inflorescence meristems^7–9^. *JOINTLESS* (*J*) is a MADS-box gene located on chromosome 11^4, 10^ and is expressed in the inflorescence meristems^9^ and at very early stages of AZ formation^11^. The *j* mutant is a null allele, carrying a large deletion in the 5′-end of the coding sequence including the first 33 bp of the MADS domain^4^.

The J protein interacts physically with several members of the MADS-box family of transcription factors^12^. Recent advances focused on the formation of a complex between J and two of them, MACROCALYX (MC)^13^ and SlMBP21, whose down-regulation causes incomplete pedicel AZ development^11^. It was therefore suggested that a MADS-box protein complex comprising, at least, J, MC and SlMBP21 regulates pedicel AZ development in tomato^11, 14^. Similar to *J*, *MC* suppression also conditions pleiotropic effects including inflorescence indeterminacy and a leafy calyx^13, 15, 16^. The association of the lack of AZ with inflorescence and floral organ phenotypes in the *j* and *mc* mutants suggests that pedicel AZ formation may be an indirect effect of the genetic network regulating inflorescence architecture in tomato^7, 17^. Recently, the *j* mutant has been instrumental for identifying genes expressed during formation of the AZ. Transcription factors that regulate meristem functions such as the tomato homolog of *WUSHEL* (*LeWUS*), *GOBLET* (*GOB*), *LATERAL SUPPRESSOR* (*Ls*) and *Blind* (*Bl*)^13, 18^ were found associated with AZ formation. These data provided molecular support to the similarities between AZ layers at pre-abscission stage and meristematic cells and suggested an ancient signalling system to assure indeterminate cell maintenance in the AZ^19^.

Despite that the characterization of *j* has brought new insights into the molecular mechanisms controlling AZ formation, its use for breeding was a failure. Introgression of *j* allele led to great foliation and low yield. In contrast, *j-2* alleles were successfully introgressed, since they caused the absence of pedicel without major drawback^7^. The *j-2* mutation was first discovered in a wild species, *S. cheesmaniae* (LA0166), originating from the Galapagos Island^20^ and later in a commercial field^21^. Beside the jointless pedicel character, *j-2* is associated with bifurcate inflorescences producing an abnormally large number of flowers and conversion of sepals to leaf like structures^21^. Complementation tests showed that *j-2* and *j* are non-allelic^22^; *J-2* was mapped to the centromeric region of chromosome 12^5, 23, 24^ but the causal gene remains to be identified.

In this study, we conducted a combination of physical mapping and gene expression analysis to identify positional candidate genes associated with *j-2* phenotype. The availability of a new version of the tomato genome and meta-analysis of expression data allowed us to pinpoint key candidates. Targeted knockouts, using CRISPR/Cas9 genome editing method, validated our hypothesis and demonstrated that *J-2* encodes the MADS-box protein SlMBP21. Molecular analysis of *J-2* natural alleles revealed two independent loss-of-function mutants. One allele results from a transposon insertion that may have co-suppressed *in cis* the expression of *SlMBP21*. The second allele results from a stop codon mutation that leads to a truncated protein. Detailed phenotypic analyses of *j j-2* double mutant revealed that *J* is epistatic to *J-2*. The role of *J-2* in AZ formation, in leafy sepals and in inflorescence branching is discussed.

## Results

### *Jointless-2* accessions display light intensity-dependent penetrance

**Figure 1:**
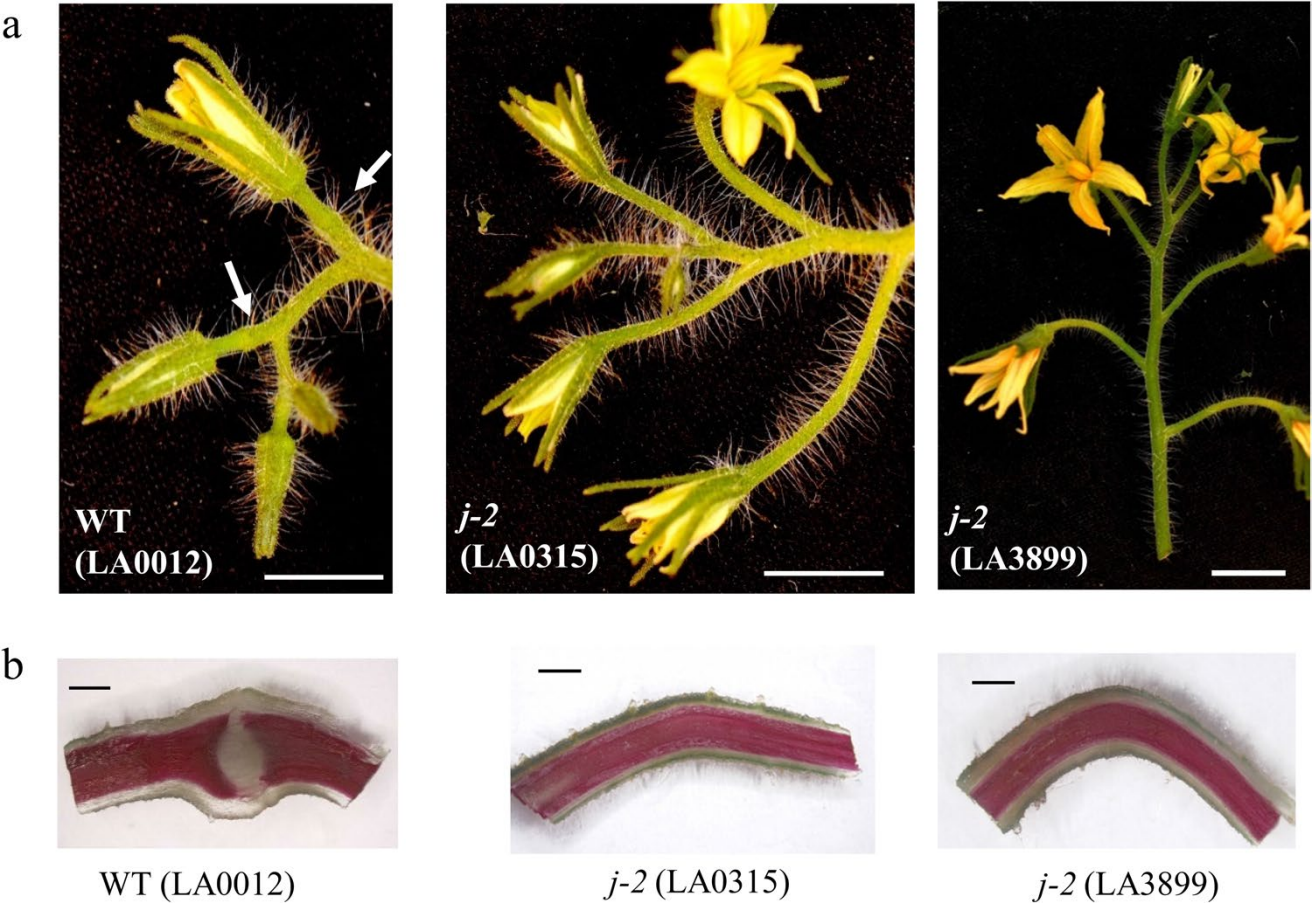
Phenotypes of the *j-2* tomato mutants. (**a**) Inflorescences of WT var. Pearson (LA0012), *j-2* (LA0315) and *j-2* (LA3899) mutants are shown. White arrows indicate abscission zone in WT plants. Scale = 1 cm. (**b**) Longitudinal sections of flower pedicels stained with phloroglucinol, showing the presence of the AZ in WT and not in the *j-2* mutants. Scale = 2 mm.

Two allelic *j-2* mutants (LA0315 and LA3899) were obtained from the Tomato Genetics Resource Center (TGRC). Both mutants are in determinate genetic backgrounds carrying a mutation in the *SELF PRUNING* (*SP*) gene: LA0315 is in Pearson (LA0012) background and LA3899, in addition of *sp* and *j-2* mutations already described in its Ohio8245 parent^25^, carries mutations *B* and *u* accounting for orange and uniform ripening fruits, respectively. Both *j-2* alleles suppress the development of the AZ at the flower pedicels characteristic of WT plants. This phenotype was constant in the LA0315 mutant, where the flower pedicels were also longer than in WT Pearson (Fig. 1a). The LA3899 mutant lacked flower pedicel AZ (Fig. 1a,b) but in some cases formed knuckle-like structures that externally looked like the AZ but did not contain the separation cell layers (Supplementary Fig. 1a,b,c,d). This phenotype occurred more obviously under low light intensity and in the proximal flowers of the inflorescences, but was much weaker under high light intensity and in distal flowers (Supplementary Fig. 1d, e). Occasionally, small leaves were observed in the inflorescences of the LA0315 mutant but were then at the base of the inflorescence and not produced after the flowers. Branching was also observed, giving the *j-2* inflorescence a biparous instead of uniparous structure, and flowers exhibited longer sepals (Supplementary Fig. 2a). These leafy sepals remained attached to the fruit during its development and maturation (Supplementary Fig. 2b). Branched inflorescences were also observed in the LA3899 mutant under high light intensity whereas under low light intensity, branching of the inflorescence was less frequent (Supplementary Fig. 1d, e). Altogether, our phenotyping, in different growing conditions, points out that those *j-2* mutations are pleiotropic and display different penetrance, depending on light conditions. These data also suggest that *j-2* mutations in LA0315 and LA3899 are likely to be different.

### Identification of candidate genes associated with *j-2* locus

The *j-2* mutation was discovered in a wild species, *S. cheesmaniae* (LA0166), originating from the Galapagos Island in Ecuador and is recessive^24^. Description from the TGRC indicates that *j-2* mutation in LA0315 line derived from *S. cheesmaniae*, whereas *j-2* in LA3899 seems to have appeared as a spontaneous mutation. In order to check that both *j-2* mutants used in this study were affected in the same gene, a complementation test was performed. All the F1 offspring of the LA0315xLA3899 cross showed the jointless phenotype, confirming that the *j-2* mutations in LA0315 and LA3899 stocks were indeed allelic (Supplementary Fig. 3a). To exclude the possibility that the absence of the AZ resulted from the inactivation of *J* (*Solyc11g010570*), we analyzed the expression of *J* in lines LA0315 and LA3899, harboring *j-2* alleles. As expected, the expression of *J* was not different than in WT plants (Supplementary Fig. 3b).

**Figure 2:**
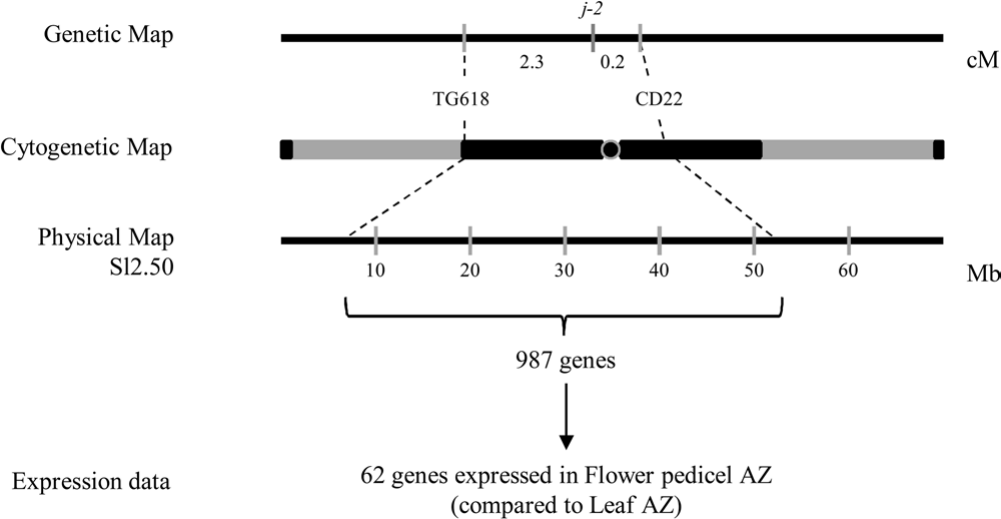
Method used to identify positional candidate genes for *j-2*. Representation of TG618 and CD22 markers on the genetic, cytogenetic and physical maps on chromosome 12 of the tomato genome (modified from Budiman M.A. *et al*.^24^). Total numbers of annotated genes in TG618-CD22 genetic interval, and of selected genes particularly expressed in flower pedicel abscission zone are also displayed. See the complete list in Supplementary Table 1.

**Figure 3:**
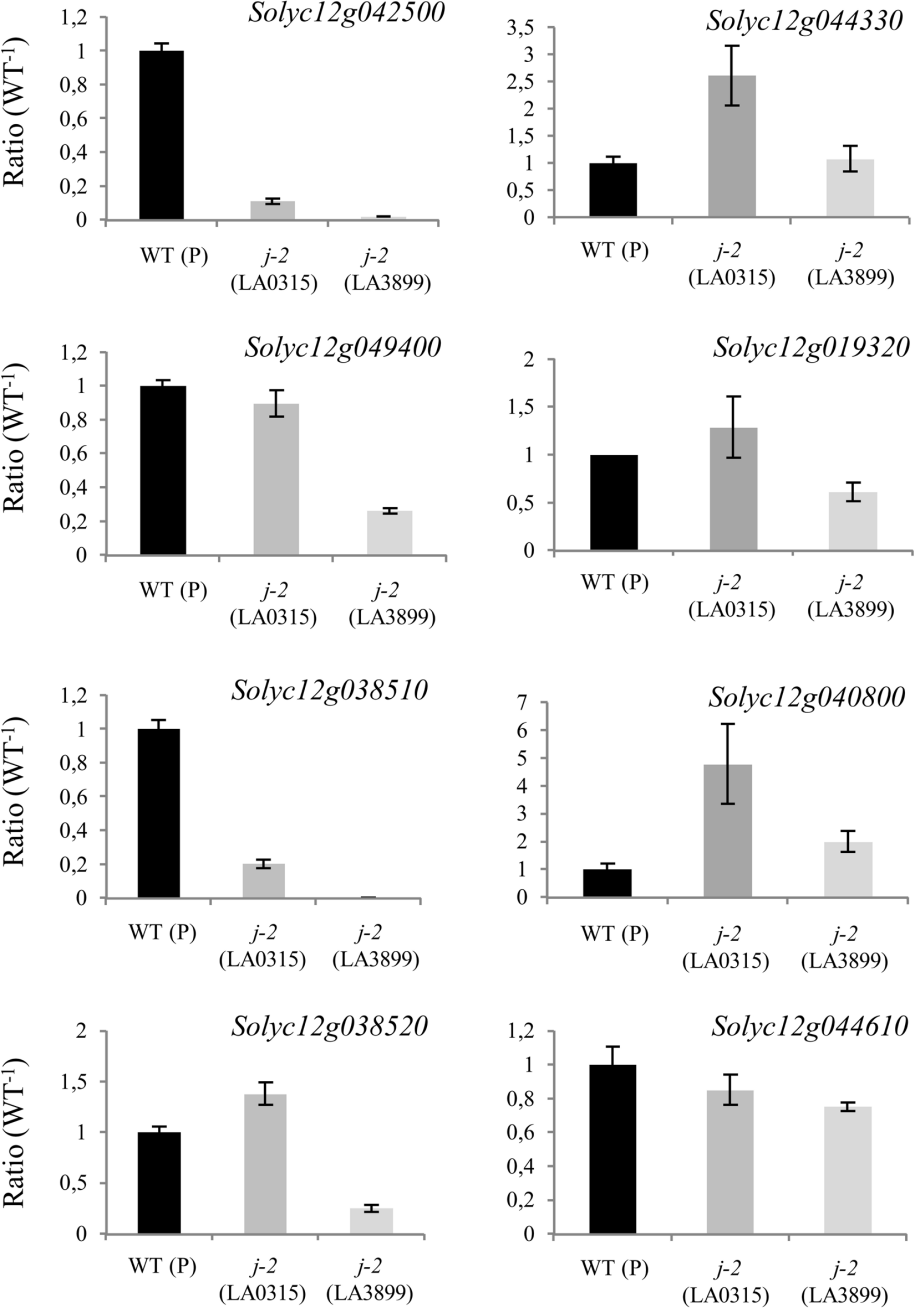
Expression levels of candidate genes in *j-2* mutants, relative to WT. The selected genes are expressed in the AZ and located in TG618-CD22 genetic interval. WT (P, Pearson) and *j-2* flower pedicels were used as plant material. Bars = SDVE.

A restriction fragment length polymorphism (RFLP) analysis mapped *j-2* in a 2.4 cM interval between CD22 and TG618 markers on chromosome 12^24^. These two markers delimited a large heterochromatic region from each side of the centromere, making the precise localization of the *J-2* locus difficult to predict. The complete sequencing and annotation of the tomato genome allowed us to re-examine the physical mapping and to identify positional candidates that map between CD22 and TG618 markers. Version *Sl*.2.50 of the genome predicted a total of 987 genes in *J-2* genetic interval (Fig. 2, Supplementary Fig. 4a). To reduce this large list to a minimum number of candidate genes, we carried out a meta-analysis of RNAseq data searching for genes that are highly expressed in the flower pedicel AZ compared to leaf AZ^26^. Among the 987 genes that physically mapped to *J-2* interval, 62 were found as differentially expressed in the flower pedicel abscission zone (FAZ) compared to the leaf abscission zone (LAZ). We selected 8 of them, based on the expression level (average read depth on FAZ > 50) and the function of the encoded proteins (transcription factors, components of hormonal pathways, developmental genes) (Fig. 2, Supplementary Table 1). Among the 8 selected candidates, the gene that showed the most significant differential expression between LAZ and FAZ (Log2 ratio = −8,41) was a MADS box transcription factor (*MADS11*, *Solyc12g038510*). The 7 other candidate genes encode transcription factors or hormone signaling regulators: a gibberellin-regulated protein (*Solyc12g042500*), an aquaporin (*Solyc12g044330*), a jasmonate ZIM domain-containing protein (*Solyc12g049400*), a multidrug resistance protein (*Solyc12g019320*), a C2 domain-containing protein (*Solyc12g040800*), a SQUAMOSA promoter binding protein (*Solyc12g038520*) and a MYB transcription factor (*Solyc12g044610*). To further reduce the list of candidate genes, we compared their expression levels in WT and *j-2* mutants (Fig. 3). cDNA was prepared from pedicel segments harvested where the AZ formed (WT) or should have formed (*j-2*), at the time of flower anthesis. The transcript levels of two candidate genes (*Solyc12g042500* and *Solyc12g038510*) were reduced to a great extend in both *j-2* mutants whereas expression of the other six was similar in WT and in one or the other *j-2* mutant. Based on these expression analyses, we reduced the list of candidate genes to *Solyc12g042500* and *Solyc12g038510* genes. *Solyc12g038510* was previously annotated as *SlMBP21* and associated with AZ development^11^. Nevertheless, the authors excluded *SlMBP21* as a candidate causing *j-2* mutation, because the assembly of the tomato genome (version *Sl*.2.40) localized *Solyc12g038510* outside the TG618-CD22 interval (Supplementary Fig. 4b). The second gene, *Solyc12g042500*, has never been associated with AZ formation.

### A Rider retrotransposon insertion underlines *jointless-2* allele in line LA3899

We started by investigating whether *J-2* locus corresponds to the *Solyc12g042500* gene. We sequenced *Solyc12g042500* in WT and LA0315 and LA3899 lines harboring two different alleles of *j-2*. We did not detect any mutation associated with *j-2* alleles in *Solyc12g042500*.

**Figure 4:**
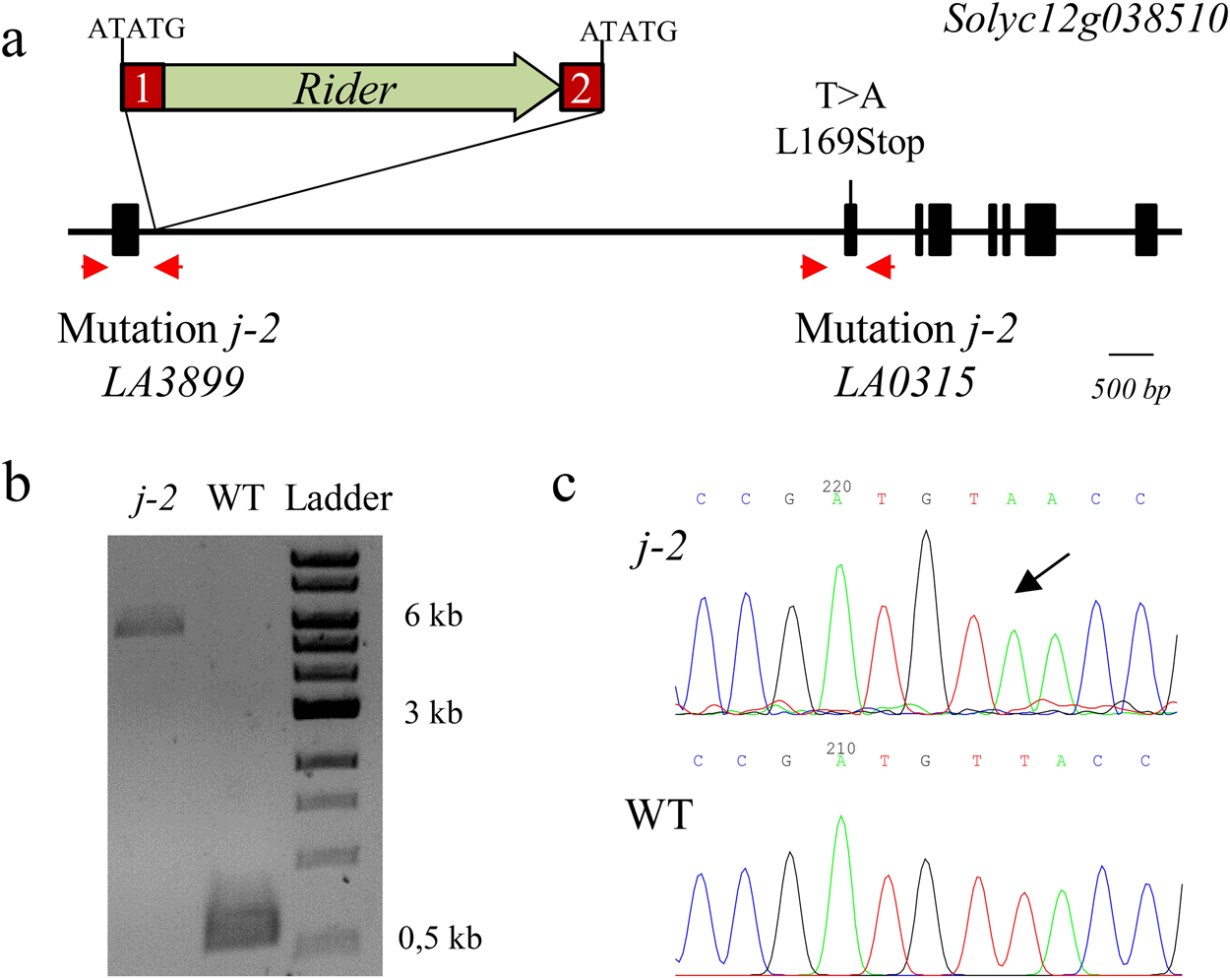
Mutations found in the *j-2* mutants (LA0315 and LA3899). **(a**) Insertion of a Transposable Element (*Rider*) in LA3899 and base substitution introducing a stop codon in LA0315. (**b**) Size of the fragments (5460- and 587-bp) amplified between the first exon and the first intron in *j-2* (LA3899) and WT genomic DNA (compared to 1 kb DNA Ladder). The largest band was extracted and cloned in the pGEM-T Easy vector for sequencing. (**c**) Sequencing results of WT and *j-2* (LA0315) PCR products amplifying the second exon of the gene.

Then we analyzed DNA sequence of *Solyc12g038510*, which codes for a MADS-box protein. We designed primers to amplify different regions of *Solyc12g038510* (five segments between the 5′UTR and the 3′UTR) and amplified fragments in both *j-2* lines and WT. Interestingly, in line LA3899, the 587-bp expected fragment from the first PCR (5′UTR-first intron) was not amplified, but a longer fragment was obtained. Sequence analysis showed that a retrotransposon of the *Rider* family was inserted 23 bp downstream of first exon’s end, suggesting that the insertion of the retrotransposon is the cause of the jointless phenotype in this line (Fig. 4a,b, Supplementary Fig. 5a and b). Similar to the *Rider* retrotransposon detected in the *sun* locus^27^, *Rider* in *j-2* (LA3899) was flanked by two 398-bp identical long terminal repeats (LTR 1 and 2) and a 5-bp target site duplication (TSD) sequence ATATG (Fig. 4a, see Subtext 1). Transposable elements constitute a large fraction of plant genomes and are subjected to epigenetic modification affecting the expression of linked genes, notably by the genomic spreading of DNA methylation. Because insertion of the *Rider* transposon in *Solyc12g038510* in line LA3899 was detected in a noncoding sequence (first intron) and correlated with no expression of the gene, we hypothesized that *Rider* insertion caused spreading of DNA methylation to nearby *Solyc12g038510* gene in *j-2* (LA3899) line. We examined the DNA methylation status of the *Solyc12g038510* locus using immunoprecipitation of DNA fragments with a 5′-methylcytosine antibody (MeDIP) in combination with quantitative PCR analysis. The methylation state was assayed in the AZ of pedicel samples from WT and LA3899 line. As expected, we observed strong methylation of *Rider* retrotransposable element, independently of the genetic context (Supplementary Fig. 6). We searched for potential spreading of DNA methylation from *Rider* to *Solyc12g038510* neighboring introns and exons. The first exon and the first intron, immediately flanking the *Rider* insertion, were hypermethylated in the *j-2* (LA3899) line compared to WT (Supplementary Fig. 6). Altogether these data suggest that spreading of DNA methylation from *Rider* insertion may be the cause of the non-expression of *Solyc12g038510* gene in line LA3899.

As *j-2* mutations in LA3899 and LA0315 are allelic, we expected to identify a disruption of *Solyc12g038510* in line LA0315 too. Sequencing of *Solyc12g038510* in line LA0315 revealed a single base substitution (T > A) in the second exon causing a premature stop codon in the protein sequence (Fig. 4c, Supplementary Fig. 5c). Consistent with this, the same stop codon mutation was also found in the LA0166 line from Galapagos that served as genetic background to introgress *j-2* allele in LA0315.

### CRISPR/Cas9-induced deletions in *Solyc12g038510* lead to jointless phenotype

**Figure 5:**
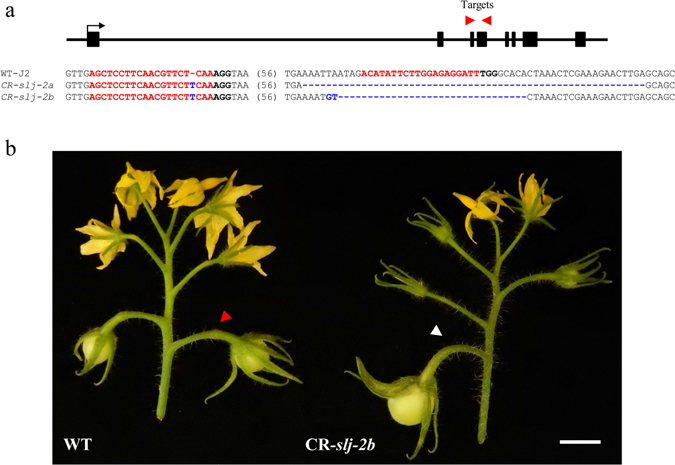
A CRISPR/Cas9-engineered mutation in *J-2* gene abolishes development of the flower AZ. (**a**) Two sgRNAs (red arrows) were designed to target the second and third exons of the *J-2* gene. Types of DNA lesions generated in two T1 CRISPR/Cas9 *J-2* (CR-*slj-2*) plants and identified by PCR genotyping from loss-of-function alleles CR-*slj-2a* and *b* (bold red: sgRNA targets, bold black: PAM site, dashes: deleted nucleotides, blue letters: inserted nucleotides). (**b**) Representative inflorescence of WT and CR-*slj-2b* mutant. Arrows indicate the AZ in WT (red) or the expected place of AZ in CR-*slj-2b* (white).

To unambiguously validate the identity of the *Solyc12g038510* gene as *J-2*, we created loss-of-function mutations using CRISPR/Cas9 technology. Two single-guide RNAs (sgRNAs) were designed to target the third and fourth exons of *Solyc12g038510* (Fig. 5a). Both constructs led to deletions in *Solyc12g038510* that correlated with absence of AZ. For deep analyses, we chose an allele (CR-*slj-2b*) where an out-of-frame deletion had introduced a premature stop codon. Like the natural *j-2* mutants and the *SlMBP21-AS* RNAi transgenic plants^11^, CR-*slj-2* mutants displayed pedicels lacking AZ (Fig. 5b). Interestingly, leafy-like sepals and elongated fruits were not observed in CR-*slj-2* lines as earlier described in *j-2* mutant (LA0315), suggesting that these characters are likely the consequence of linkage drags during introgression of *j-2* alleles into cultivated tomatoes. Similarly, only uniparous inflorescences were obtained in *CR-slj-2* lines, demonstrating that the inflorescence branching, observed in LA0315 plants or in LA3899 plants growing under high light conditions, is *j-2* independent. We therefore analyzed whether the knuckle-like structures observed in *j-2* plants (LA3899) growing under low light conditions were the result of residual *J-2* expression. RT- qPCR analysis in pedicels of the first flower of the inflorescence of *j-2* (LA3899) growing under low and high light intensity showed that the knuckle-like phenotype is not correlated with *J-2* expression but most probably due to the activity of other genes involved in AZ formation (*J* or *MC*) (Supplementary Fig. 7).

### Expression analysis of *J-2* and interacting genes

We examined *J-2* expression pattern by *in situ* hybridization in WT and *j-2* mutants. *J-2* mRNA was detected in flower meristems of WT and *j-2* line harboring the stop codon (LA0315) but not in line LA3899 harboring *Rider* retrotransposon. At later stage, *J-2* transcripts were detected in the region of the AZ in the WT only (Supplementary Fig. 8). These results suggest that *J-2* is expressed during early inflorescence development and is later required for the formation of the AZ. Similar results were described by Liu *et al*.^11^ analyzing the *SlMBP21* gene expression pattern.

**Figure 6:**
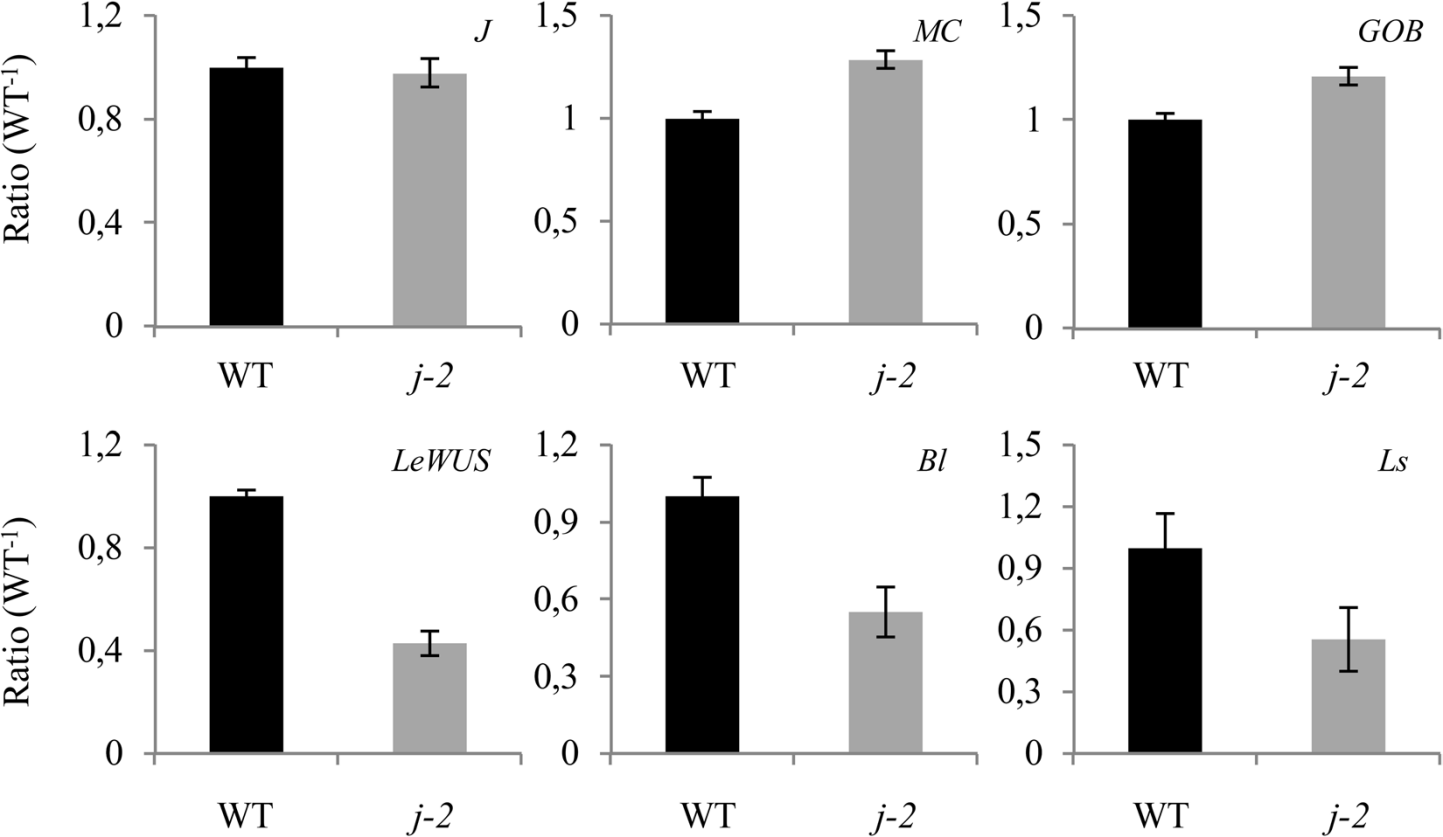
Expression levels of genes involved in the formation of the abscission zone of the flower pedicel in *j-2* mutant (LA0315), relative to WT. *J* (*JOINTLESS*), *MC* (*MACROCALYX*), *GOB* (*GOBLET*), *LeWUS* (*WUSHEL*), *Blind* (*Bl*) and *Ls* (*LATERAL SUPPRESSOR*). Bars = SDVE.

Since SlMBP21 was previously shown to form, with J and MC, a higher order protein complex mediating AZ formation^11^, we investigated whether *j-2* mutations affected the expression of *J*, *MC* and downstream genes (Fig. 6). Expression of *J* and *MC* was no different in the *j-2* mutant (LA0315) than in WT and genes involved in AZ formation including *Ls*, *LeWUS* and *Bl* were specifically down-regulated in the AZ of *j-2* mutant. By contrast, *GOB* did not shown significant changes.

**Figure 7:**
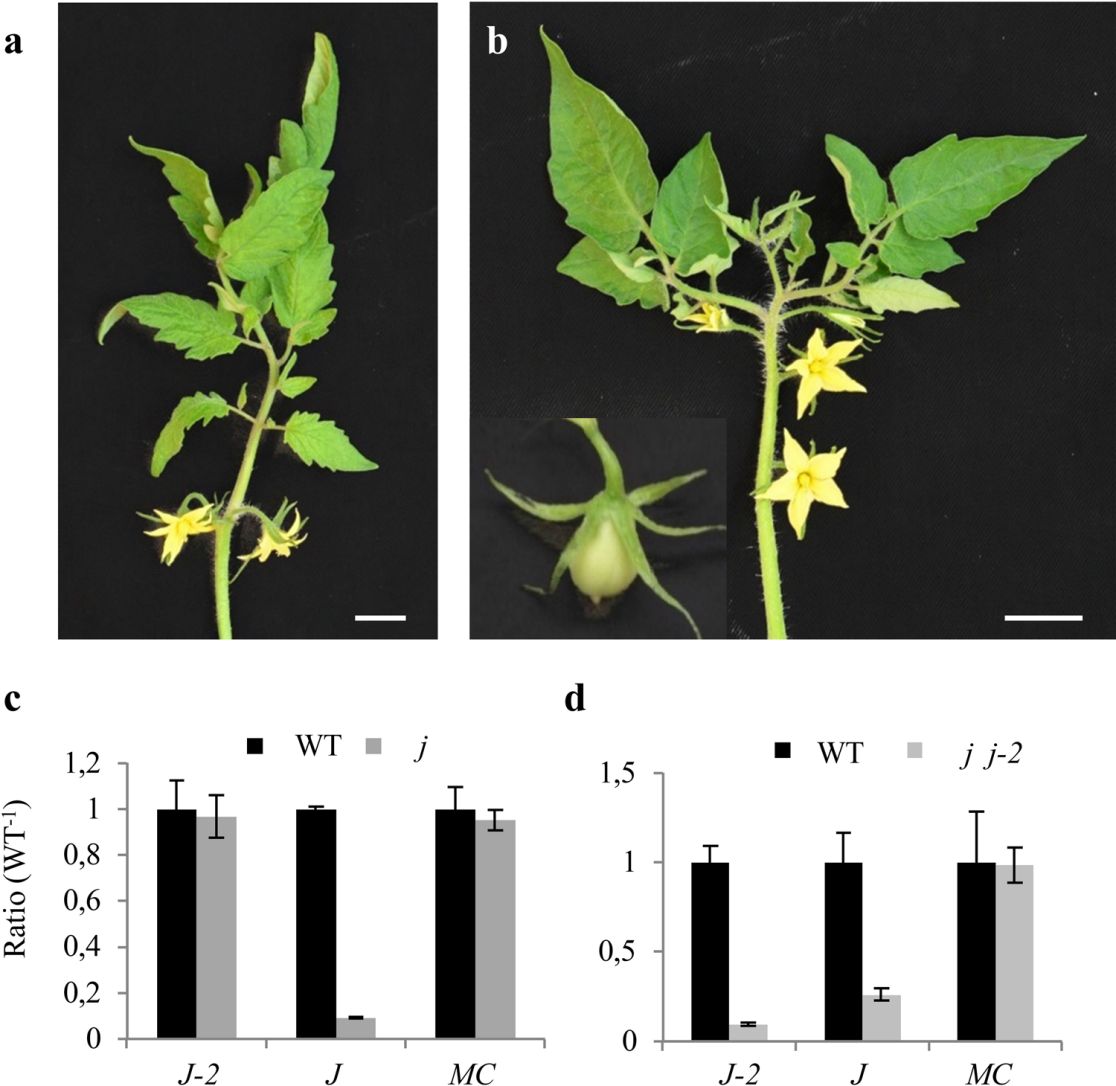
Phenotype of single *j* mutant and double *j j-2* mutant. (**a**) Leafy inflorescence of *j* mutant. (**b**) Leafy, branched inflorescence and elongated fruits in the *j j-2* line obtained by Philouze^22^. Scale = 1 cm. (**c**) Expression levels of *J-2*, *J* and *MC* in *j* mutant, relative to WT (var. Gardener). (**d**) Expression levels of *J-2*, *J* and *MC* in *j j-2* mutant, relative to WT (var. Pearson). *J-2* (*SlMBP21*), *J* (*JOINTLESS*), *MC* (*MACROCALYX*). Bars = SDVE.

The *j* mutant, in addition of AZ defect, exhibits leafy inflorescences due to resurgence of vegetative meristems in the iterative process making the sympodial inflorescence^4, 7, 9^ (Fig. 7a). By contrast, *j-2* mutants did not show the same phenotype. In order to further analyze the interactions between *J* and *J-2* genes, we phenotyped double *j j-2* mutants produced by Philouze^22^ in a determinate (*sp*) background. Sequencing analysis confirmed that this line contains the *j-2* allele with the stop codon (as that from LA0166 and LA0315). The double mutants formed leafy inflorescences with jointless flowers and hence had the same phenotypes than *j* mutant, indicating that *j* was epistatic to *j-2* (Fig. 7b). Fruits were also elongated as observed in the distal part of the truss of the *j-2* (LA3899) and sometimes in *j-2* (LA0315) mutants (Supplementary Fig. 1b, 2a). Expression data confirmed down-regulation of *J* in the single *j* mutant, and of *J* and *J-2* genes in double *j j-2* mutant, whereas expression of *MC* was similar to WT in both *j* and *j j-2* mutants (Fig. 7c and d). Nevertheless, fruits of CR-*slj-2* lines did not show elongated form (Fig. 5), indicating that this phenotype does not result from the loss of function of *J-2*.

## Discussion

More than 20 years ago, Zhang *et al*.^5^ demonstrated that the *J-2* locus, derived from the wild accession *S. cheesmaniae* (LA0166), was located on chromosome 12 and was flanked by two markers, CD22 and TG618. Even if this mutation had been extensively used during the last 60 years in the tomato processing industry, the identity of the mutated gene remained unknown. Using the tomato genome sequence and transcriptomics data from flower pedicel AZ, we identified a candidate gene encoding a MADS-box protein. Gene sequencing allowed us to confirm that *j-2* accessions (LA0315 and LA3899) from the tomato germplasm (TGRC) carry two independent mutations in the MADS-box gene *Soly12g038510*. The first allele derived from the Galapagos Island accession (LA0166) contained a single nucleotide polymorphism (SNP) introducing a stop codon; the second allele was due to a *Rider* transposon insertion in the first intron of the gene.

The *Soly12g038510* gene was previously named *SlMBP21* by Liu *et al*.^11^ and described as a new gene from the MADS-box family, involved in the formation of the flower pedicel AZ. The authors however excluded the implication of *SlMBP21* in the jointless phenotype of *j-2* mutants because the genomic interval in the previous tomato genome assembly version (*Sl2*.40 ITAG 2.3) was incorrect. Here, we genotyped two natural *j-2* mutants and produced CRISPR-Cas9 edited lines demonstrating that *SlMBP21* is the *J-2* gene.

*In situ* hybridization revealed two waves of *J-2* expression, first in the flower meristems, and later in the AZ. Branching of the tomato inflorescence was previously shown to depend on the temporal rate at which flower meristem maturate^28^. According to this temporal regulation, the increased branching in *j-2* could be interpreted by a deceleration of flower development in the mutant whereas the opposite was described for the other jointless mutant *j* ^17^. The *j* mutant indeed produces less flowers than WT and reverts to leaf production^7, 9, 29^. Interestingly, *j* mutation was epistatic to *j-2* as previously reported^22^. Given that J and J-2 proteins interact physically^11, 12^ these results suggest that these MADS-box transcription factors might balance the rate of meristem maturation in the inflorescence and have additive effects. Further analyses are required to test this hypothesis since the inflorescence phenotype was uncoupled from the lack of AZ in the CRISPR/Cas9 lines.

Breeders have introgressed *j-2* mutations from donor parents into elite lines and this might have conducted to linkage drag. Indeed the *j-2* lines obtained through CRISPR/Cas9 inactivation showed that inflorescence and flower traits might not be associated with *j-2* phenotype in LA0315 and LA3899 but might be the result of linkage drag. Now that the identity of *J-2* gene is validated, it will be possible to engineer allelic series of *Soly12g038510* and test them for AZ phenotypes as well as other side effects such as water flow through the AZ that was identified as a bottleneck for the use of *j-2* in large fruited varieties of tomato^3^.

Ito *et al*.^14^ proposed that a tetramer of MADS-box proteins including J, SlMBP21, MC and an unknown fourth partner regulates pedicel AZ formation. Interestingly, an additional gene was found by Joubert^30^ and assigned as *jointless-2* with *incomplete* action (*j-2* ^*^in*^) because absence of the AZ was only observed at the onset of fruit ripening^30^. At the difference of *j-2*, plants containing *j-2* ^*^in*^ do not show elongated peduncle and slow fruit ripening, and hence may be also suitable for breeding programs. The gene causing the *j-2* ^*^in*^ mutation remains unknown but together with *J-2* could be the key for tomato resistance to drought because it has been shown that the absence of AZ in flower peduncle increases water transport and thus allows to reduce watering during production^31^.

## Methods

### Plant material and growth conditions

Seeds were germinated in a mix of peat compost brill (85%) and clay (15%) at 20 °C. After two weeks, seedlings were transplanted into individual pots filled with a mix of peat compost brill (75%): clay (15%): perlite (10%). Experiments were carried out in growth cabinets in 16-h photoperiod, 100 (‘low’) or 200 (‘high’) µmol m^−2^ s^−1^ light fluence rate at leaf canopy level (V.H.O. Sylvania fluorescence tubes), 20 °C, 70% relative humidity. Plants were watered daily with tap water and fed every two weeks with 12-12-17 N-P-K fertiliser (Compo, Benelux N.V.).

### Genomic DNA and total RNA preparation

Tomato genomic DNA was extracted from plant inflorescences using the DNeasy Plant Mini kit (Qiagen), and pooled in equimolar ratio. Total RNA was extracted from flower pedicels (at anthesis stage) using the RNeasy Plant Mini kit (Qiagen), including DNase treatment with RNase-free DNase (Qiagen) according to the manufacturer’s instructions. First-strand cDNA synthesis was performed using 1 µg of total RNA with the SuperScript II First-Strand Synthesis System with oligo(dT)20 (Invitrogen).

### Genotyping of *j-2* natural mutants

Five different segments of the *J-2* locus were amplified using the Phusion High-Fidelity DNA Polymerase (New England Biolabs). PCR products were analyzed in 1% agarose gels in TBE buffer and visualized with ethidium bromide. Images were acquired with the imaging system E-Box UX5 version 15.11 (Vilber, Marne-la-Vallée, France). The annealing temperature for amplification was 58 °C during 34 total cycles. PCR fragments were sequenced from genomic DNA of WT and *j-2* mutants. The specific primers sequences are given in Supplementary Table S2.

### Transposon amplification and cloning

Primers located in the 5′UTR and first intron of the *J-2* locus were used to amplify the DNA segment containing the *Rider* transposon. Amplification time was set up at 4 min at 72 °C using *j-2* (LA3899) DNA as matrix and the iProof High-Fidelity DNA Polymerase (Bio-Rad). Amplified fragment was subsequently cloned into the pGEM-T Easy vector (Promega) and subjected to sequence analysis. Sequences were annotated using blast search against the SOL Genomics Network (SGN; https://solgenomics.net/).

### Quantitative PCR

Quantitative RT-PCR was performed with 20-fold diluted cDNA using SYBR Green Supermix (Bio-Rad) and gene-specific primers. The gene used as control (*Solyc03g115810*) encodes a vacuolar fusion protein (VAC)^11, 18^. Two biological replicates and two technical replicates were analyzed using the CFX384 Real-Time PCR System (Bio-Rad). Primers used in this study are listed in Supplementary Table 2.

### Methylated-DNA Immunoprecipitation-qPCR

Genomic DNA was isolated from WT and *j-2* (LA3899) mutant flower pedicels as previously described and fragmentation was performed using Diagenode Bioruptor 200 UCD-300 (30 s then off 90 s for 25 cycles, low power position). Following steps were performed using Diagenode Auto hMeDIP KIT in the SX-8G IP-Star® Compact System. Anti-5-methylcytosine antibody (NA8133D3, Merck Millipore, Diagenode) was used for precipitation. DNA was then purified using Auto Ipure kit v2 (Diagenode). MeDIP-qPCR was performed by the same methods as the RT-qPCR using the purified immunoprecipitated DNAs as templates. Primers used for MeDIP-qPCR are listed in Supplementary Table 2.

### *In situ* hybridation

Inflorescence meristems for *in situ* hybridization were dissected and fixed with 2% PBS followed by ethanol dehydration baths. *In vitro*–transcribed RNA probe for *J-2* was generated from full-length cDNA clones, and transcript was detected using standard *in situ* hybridization techniques. Primers for probe amplification are provided in Supplementary Table 2.

### CRISPR/Cas9 gene editing

CRISPR/Cas9 gene editing was performed as described previously^32, 33^. Briefly, two single-guide (sg)RNAs binding to the coding sequence of the target gene were designed using the CRISPR-P tool (http://cbi.hzau.edu.cn/cgi-bin/CRISPR)^34^. Vectors were assembled with the Golden Gate cloning system^35^. sgRNA1 and sgRNA2 were cloned downstream of the Arabidopsis U6 promoter in the Level 1 acceptors pICH47751 and pICH47761, respectively. Level1 constructs pICH47731-NOSpro::NPTII, pICH47742-35S:Cas9, pICH47751-AtU6pro:sgRNA-1, and pICH47761-AtU6::sgRNA-2 were assembled in the binary Level 2 vector pAGM4723. Binary vectors were transfected into tomato cultivar M82 by *Agrobacterium tumefaciens*-mediated transformation^36^. After *in vitro* regeneration, plants were transplanted into soil, and acclimated under transparent plastic domes in the greenhouse for 5 days. A total of 8 first-generation (T0) transgenics were genotyped for induced lesions using forward and reverse primers flanking the sgRNA target sites. PCR products were separated by gel electrophoresis and T0 plants with visible lesions were self-pollinated. The T1 generation was genotyped and PCR products were cloned into pSC-A-amp/kan vectors (StrataClone Blunt PCR Cloning Kit, Stratagene). Five clones per PCR product were sequenced using M13-F and M13-R primers.

## Electronic supplementary material

Supplementary information
