# Supplementary material for: Natural and induced loss of function mutations in SlMBP21 MADS-box gene led to jointless-2 phenotype in tomato
Source: Sci Rep. 2017 Jun 30;7:4402. doi: 10.1038/s41598-017-04556-1 (PMC5493662; doi:10.1038/s41598-017-04556-1)
Supplement: Supplementary file 1 — Supplementary information [file 41598_2017_4556_MOESM1_ESM.pdf]

SUPPLEMENTARY DATA

RESEARCH ARTICLE

Natural and induced loss of function mutations in *SlMBP21* MADS-box gene led to *jointless-2* phenotype in tomato

Maria Victoria Gomez Roldan[a+], Claire Périlleux[b+], Halima Morin[a], Samuel Huerga-Fernandez[b], David Latrasse[a], Moussa Benhamed[a], Abdelhafid Bendahmane[a,*]

[a] *Institute of Plant Sciences Paris-Saclay (IPS2), CNRS, INRA, University Paris-Sud, University of Evry, University Paris-Diderot, Sorbonne Paris-Cite, University of Paris-Saclay, 91405, Orsay, France.*
[b] InBioS, PhytoSYSTEMS, Laboratory of Plant Physiology, University of Liège, Sart Tilman Campus Quartier Vallée 1, Chemin de la Vallée 4, B-4000, Liège, Belgium.
[+] These authors equally contribute to this work.
[*]Address correspondence to abdelhafid.bendahmane@inra.fr

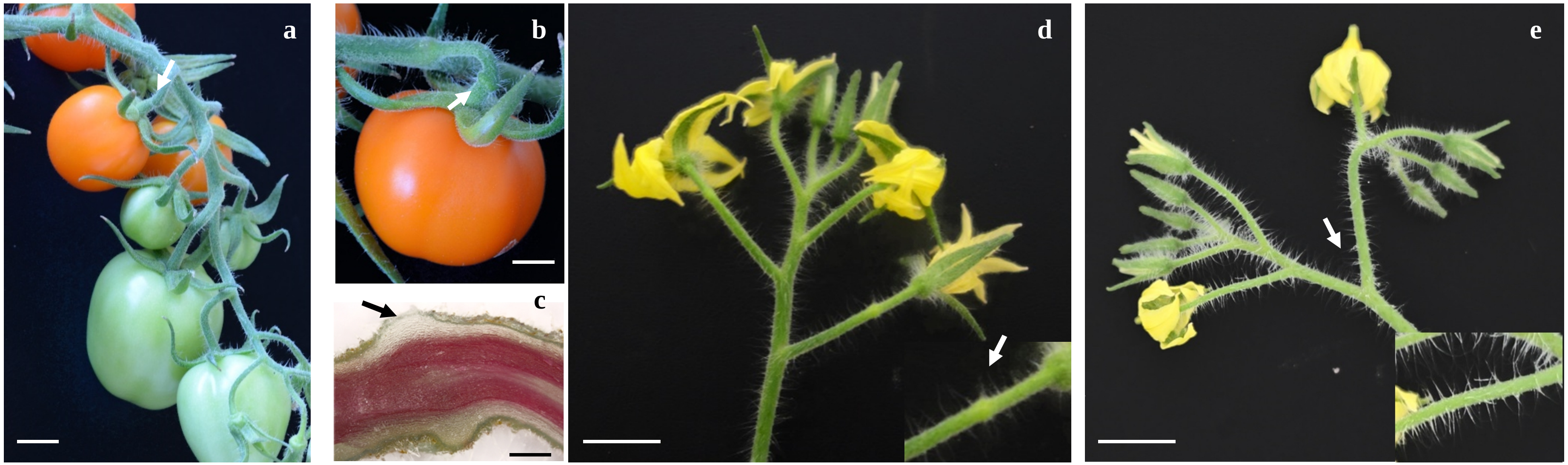

Supplementary Fig. 1. Particular phenotypes of *j-2* (LA3899) mutant. (a) Inflorescence with ripe round and unripe elongated fruits. Scale = 1cm. (b) AZ like structure on pedicels of plants growing in low light conditions. Scale = 7mm. (c) Longitudinal section of a pedicel stained with phloroglucinol. Black arrow shows the absence of AZ in the knuckle-like structure. Scale = 1mm. (d) Inflorescence of plants growing in low light conditions. The *inset* shows the formation of a knuckle-like structure on the first pedicel (white arrow). Scale = 1cm. (e) Branched inflorescence (white arrow) of plants growing in high light conditions. The *inset* shows the absence of a knuckle-like structure. Scale = 1cm

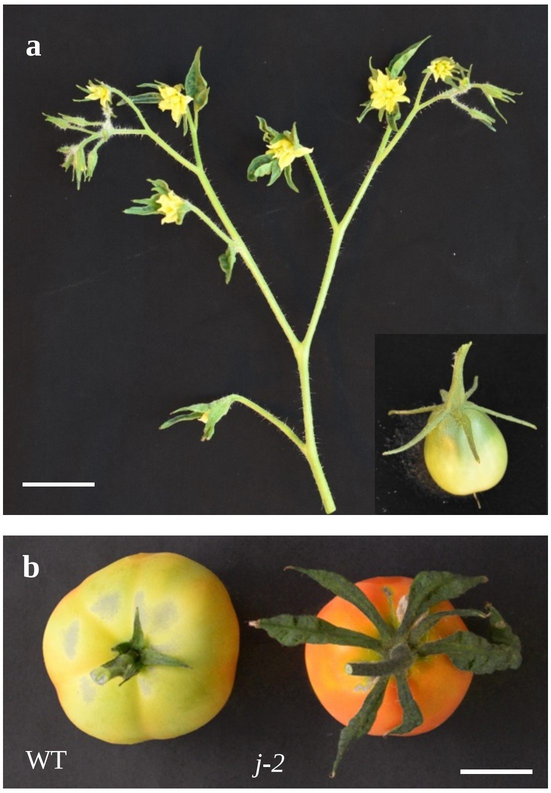

Supplementary Fig. 2. Particular phenotypes of *j-2* (LA0315) mutant. (a) Branched inflorescence structure with leafy sepals and elongated fruits. Scale = 3cm. (b) *j-2* mutant fruit showing leafy sepals compared to a WT fruit. Scale = 2cm.

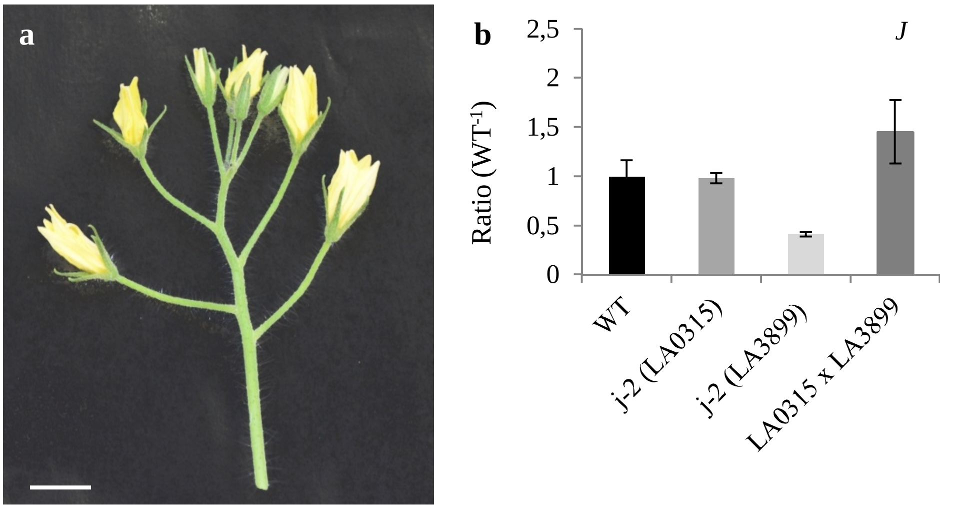

Supplementary Fig. 3. Inflorescence phenotype of *j-2* x *j-2* (LA0315 x LA3899) double mutant. (a) Inflorescence structure of F1 plants with jointless pedicels. Scale = 1cm. (b) Expression analysis of *J* in single and double mutants, relative to WT. Bar= SDVE.

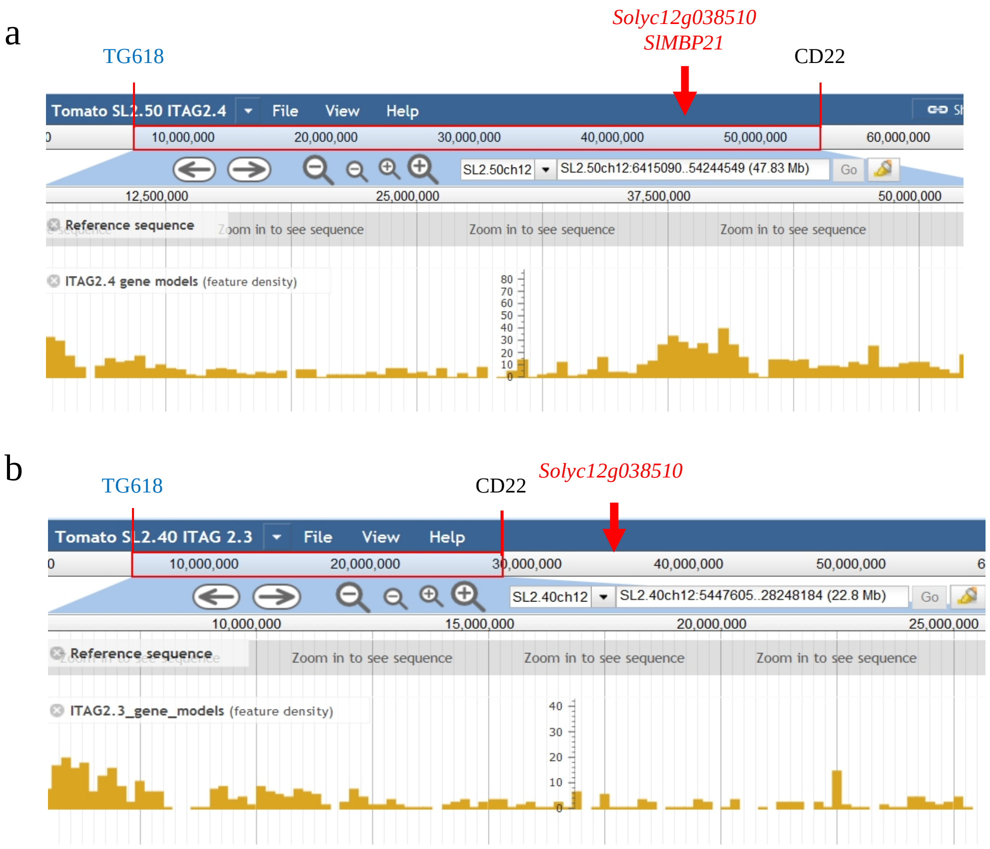

Supplementary Fig. 4. Position of TG618 and CD22 markers on chromosome 12 of the Tomato Genome. Markers TG618 (blue) and CD22 (black) in version *Sl2*.50 ITAG 2.4 (a) and *Sl2*.40 ITAG 2.3 (b). The *Solyc12g038510* gene (red) is included in the interval between the two markers according to the new version of the tomato genome from https://solgenomics.net database.

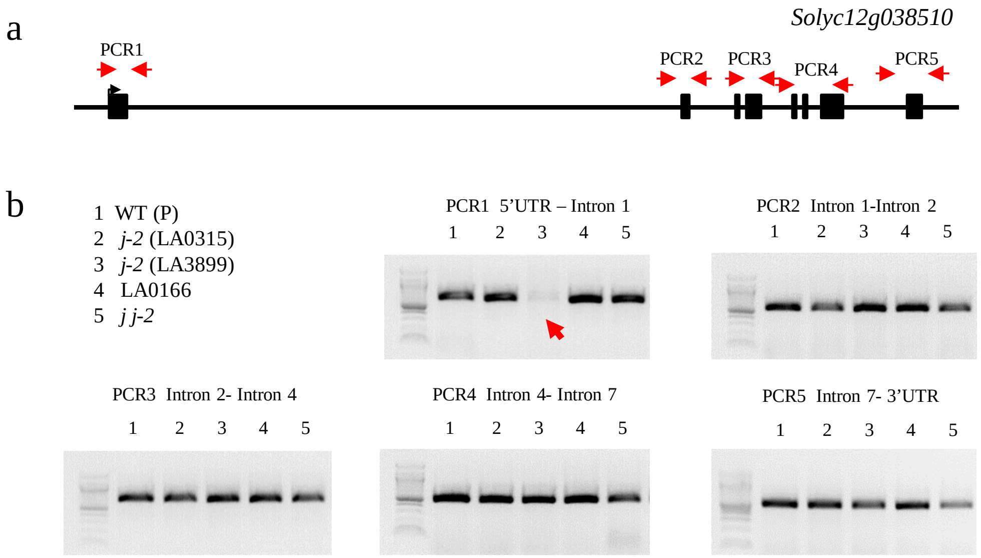

c

Translation 81 a.a.(stop codon)

MGRGRVELKRIENKINRQVTFAKRRNGLLKKAYELSILCDAEVALIIFSSRGKLYEFSSA
SSMMTTLEKYQQCSYASLDPM*PVSDTQMNYNEYVRLKARVELLQRSQRHILGEDLGTLN
SKELEQLEHQLDASLKKVRSKKTQSMLDQLADLQEKEQMLEEANKQLKNKLEESAARIPL
GLSWGNNGGQTMEYNRLPPQTTAQPFFQPLRLNSSSPQFGYNPNMGANDHEVNAATTAHN
INGFIPGWML*

Supplementary Fig. 5. Genotyping of *j-2* mutants and *j j-2* double mutant. (a) Position of the primers on the *Solyc12g038510* locus used for genotyping. (b) PCRs on genomic DNA allowing to predict the insertion of a transposon in the *j-2* mutant (LA3899). (c) Single base substitution (T >A) in the second exon causing a premature stop codon (red asterisk) in the protein sequence of *j-2* mutant (LA0315), of *S. cheesmaniae* (LA0166) and *j j-2* double mutant.

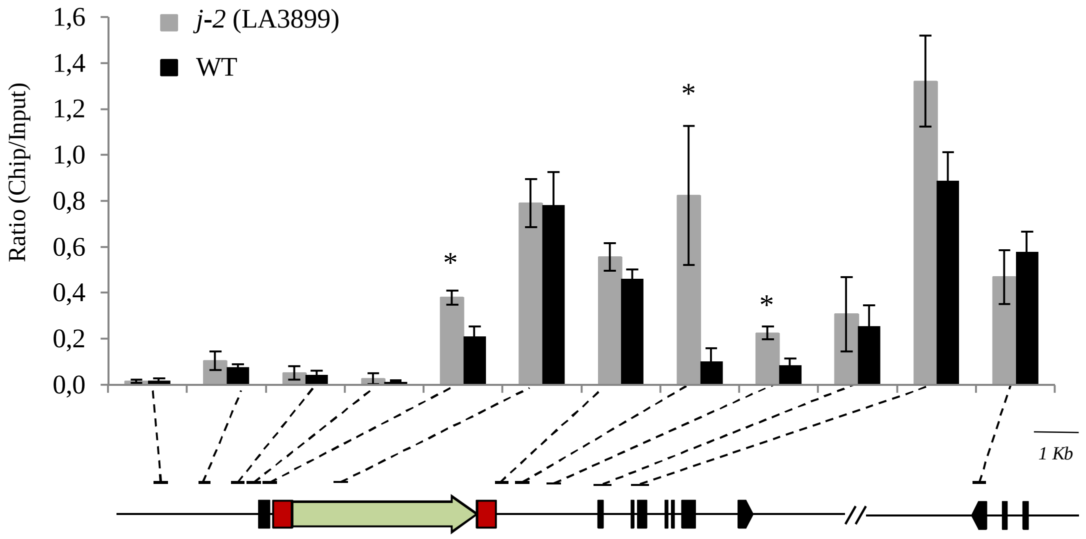

Supplementary Fig. 6. Ratio of DNA methylation levels in the *j-2* (LA3899) mutant compared with WT plants. PCR-MeDIP using anti-5meC antibody. Regions close to the *Rider* insertion were strongly methylated in *j-2* mutants. Error Bar = SDEV. * = significant enrichment (P value < 0.01).

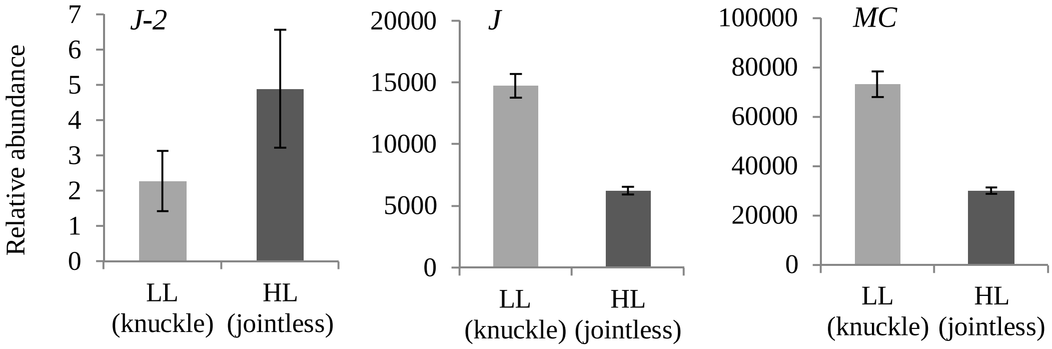

Supplementary Fig. 7. Expression of AZ genes (*J-2*, *J* and *MC*) in LA3899 flower pedicels showing or not knuckle-like structure (relative to the housekeeping gene, see Supplementary Table 2). Plants were grown under low (LL) or high (HL) light conditions. Bar = SDEV.

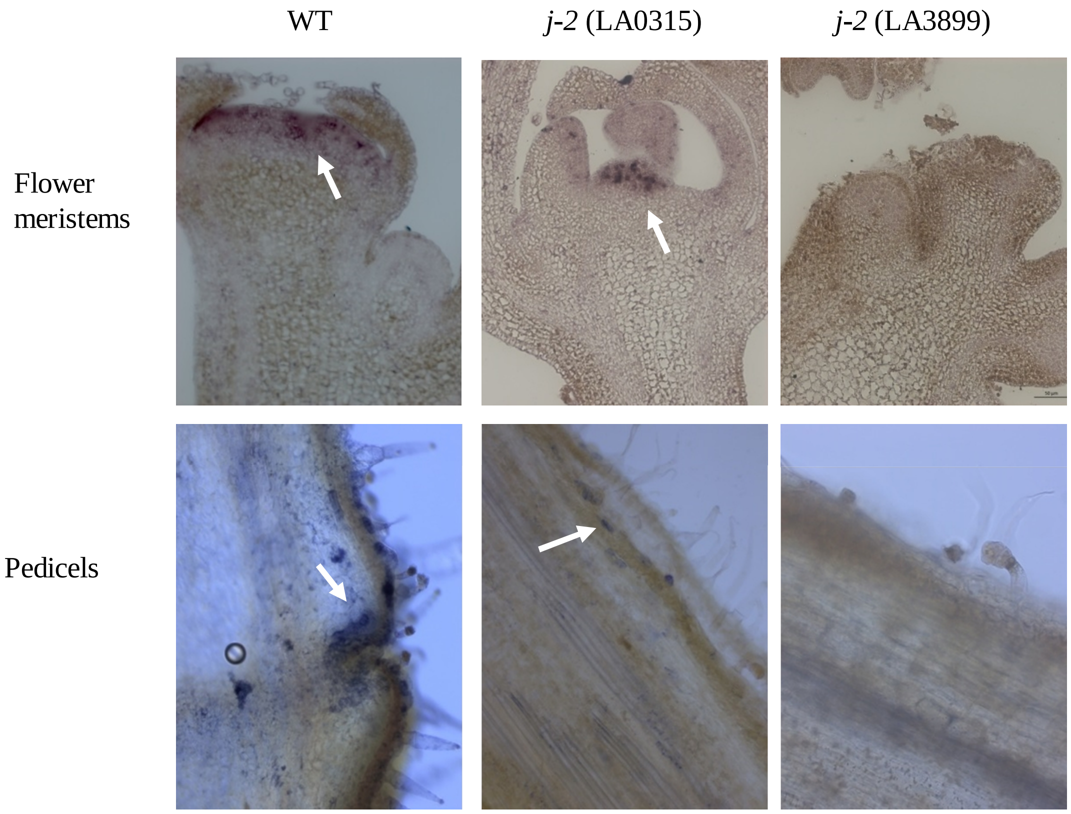

Supplementary Fig. 8. *in situ* hybridation on WT, *j-2* (LA0315) and *j-2* (LA3899) flower meristems and pedicels using *J-2* probe (CDS = 753 bp).

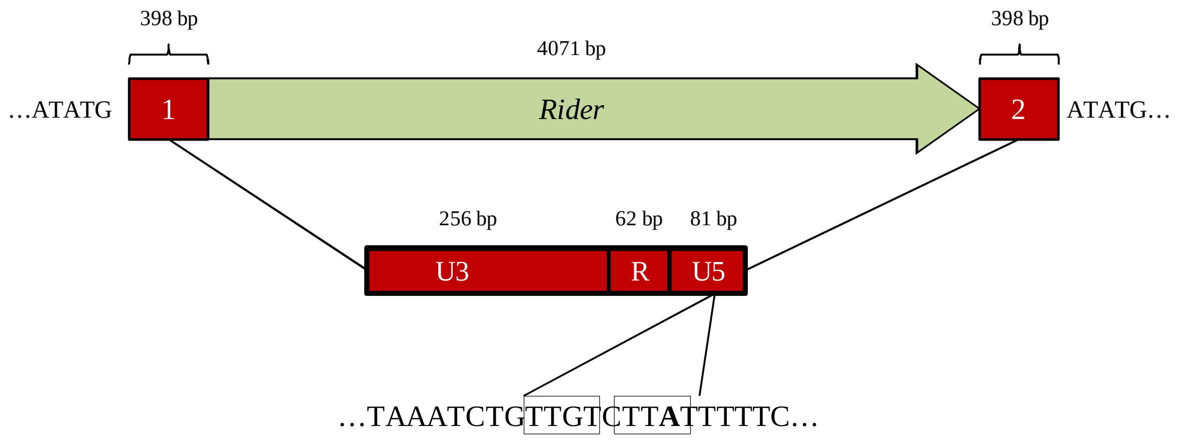

Subtext 1. *Rider* retrotransposon causing gene silencing in LA3899 accession

The *Rider* retrotransposon is a high copy *Copia* transposable element (TE) from Class I which contains in its sequence a transposase and other proteins allowing autonomous transposition. *Rider* has been shown to be ubiquitous in the tomato genome and highly correlated with the disruption of genes when it is inserted into or very close to them.

The *Rider* element inserted in *J-2* locus is 4,867 bp in length with two identical long terminal repeat (LTR, 1 and 2 in red) on each side. The LTRs are 398-bp long and flanked by the target site duplication (TSD) with a nucleotide sequence of 5 bp (ATATG). The LTR sequence contains the three classical domains called U3 (the promoter), R (the polyadenylation signal), and U5 (the termination of synthesis of element RNA). The *Rider* in *J-2* locus (LA3899) contains a mutation in the U5 domain, one copy of the two TTGT boxes was mutated to TTAT in both LTR as observed in the *sun* locus. This seems to indicate a putatively read-through transcription of Rider on *j-2* locus (LA3899).

The rest of the *Rider* sequence (in green) encodes capsid-like proteins (CP), protease (PR), integrase (INT), reverse transcriptase (RT), and RNase-H (RH).

Supplementary Table 1. List of genes on chromosome 12 found in the TG618-CD22 genetic interval and differentially expressed in the flower pedicel abscission zone (FAZ) compared to the leaf abscission zone (LAZ). Average read depth (Avg RD) expression data were obtained from Sundaresan *et al.* [26]

Gene name	Gene Description	Avg RD FAZ	Avg RD LAZ	Log2(Ratio)
Solyc12g042500	Gibberellin-regulated family protein	4484,99	960,85	-2,23
Solyc12g044330	Aquaporin	2034,81	177,26	-3,54
Solyc12g049400	Protein TIFY 3B	1890,25	58,60	-5,04
Solyc12g019320	Multidrug resistance protein mdtK	847,36	121,39	-2,81
Solyc12g019740	Thioredoxin family protein	573,13	230,31	-1,32
Solyc12g038510	MADS box transcription factor 11	461,88	2,01	-8,41
Solyc12g044420	Avr9/Cf-9 rapidly elicited protein 146	377,11	65,83	-2,53
Solyc12g039030	Photosystem Q	285,49	49,92	-2,53
Solyc12g042770	Chloroplast post-illumination chlorophyll fluorescence increase protein	259,19	86,69	-1,58
Solyc12g042580	Blue copper-like protein	220,03	68,39	-1,70
Solyc12g040800	C2 domain-containing protein	209,78	5,39	-5,32
Solyc12g017530	Unknown Protein	192,84	16,00	-3,60
Solyc12g019550	Unknown Protein	148,39	16,27	-3,22
Solyc12g044630	Profilin	131,55	34,83	-1,92
Solyc12g038520	Squamosa promoter binding protein 3	116,54	18,07	-2,73
Solyc12g039070	Strictosidine synthase family protein	112,47	6,16	-4,19
Solyc12g044280	Photosystem I reaction center subunit VI, chloroplastic	111,40	21,36	-2,40
Solyc12g042730	Unknown Protein	92,06	6,65	-3,83
Solyc12g044610	Myb transcription factor	88,90	42,14	-1,08
Solyc12g021280	Protein kinase	78,72	33,94	-1,21
Solyc12g036170	Unknown Protein	66,69	8,82	-2,94
Solyc12g049540	Polyphosphoinositide phosphatase	64,60	30,03	-1,11
Solyc12g049250	Unknown Protein	56,09	3,19	-4,19
Solyc12g044820	Multidrug resistance protein ABC transporter family	55,49	11,46	-2,30
Solyc12g045020	Cytochrome P450	47,15	12,20	-1,97
Solyc12g036330	Receptor-like protein kinase At3g21340	36,65	15,94	-1,20
Solyc12g038080	Photosystem II CP43 chlorophyll apoprotein	35,15	12,88	-1,53
Solyc12g062200	Unknown Protein	29,79	5,56	-2,47
Solyc12g049280	NAD-dependent epimerase/dehydratase	27,07	13,13	-1,16
Solyc12g036390	Calmodulin-binding protein-like	22,65	3,99	-2,54
Solyc12g040640	Beta-glucosidase	20,97	2,00	-3,67
Solyc12g049630	Unknown Protein	20,33	4,37	-2,24
Solyc12g017600	Unknown Protein	20,03	6,73	-1,63
Solyc12g042110	Unknown Protein	17,18	4,66	-2,00
Solyc12g038800	Unknown Protein	17,10	7,01	-1,67
Solyc12g082730	Kinesin-like calmodulin binding protein	14,90	4,44	-1,84
Solyc12g027760	Unknown Protein	14,10	5,87	-1,28
Solyc12g026470	Unknown Protein	12,72	4,78	-1,44
Solyc12g042520	Gibberellin-regulated family protein	12,17	2,67	-2,32
Solyc12g019640	ATP-binding cassette transporter	10,22	3,57	-1,63
Solyc12g049360	Receptor-like protein kinase At3g21340	8,73	2,40	-2,45
Solyc12g042100	Unknown Protein	7,68	1,11	-3,32
Solyc12g044260	D-isomer specific 2-hydroxyacid dehydrogenase	6,52	1,63	-3,36
Solyc12g042880	cDNA clone J013073D14 full insert sequence	6,17	1,45	-2,68
Solyc12g049550	GDSL esterase/lipase 5	5,88	2,40	-2,22
Solyc12g077360	Unknown Protein	5,34	1,39	-2,58
Solyc12g036320	Serine/threonine protein kinase B	4,97	2,20	-1,65
Solyc12g077650	30S ribosomal protein S12 chloroplastic	4,71	1,83	-1,32
Solyc12g044680	Unknown Protein	4,45	2,00	-1,32
Solyc12g038570	Unknown Protein	4,31	1,59	-1,58
Solyc12g019630	Unknown Protein	4,27	1,00	-2,58
Solyc12g077370	CBL-interacting protein kinase 6	3,93	1,66	-1,85
Solyc12g019270	Ulp1 protease family C-terminal catalytic domain containing protein	3,80	1,00	-2,32
Solyc12g036550	Hypothetical chloroplast RF1	3,63	1,76	-1,17
Solyc12g049350	Myb 12 transcription factor	3,24	1,32	-2,25
Solyc12g036810	Kinesin-like protein 73641-79546	3,01	1,31	-2,17
Solyc12g040870	Nudix hydrolase 4	2,90	1,67	-1,46
Solyc12g017380	Solute carrier family 22 member 7	2,49	1,83	-1,17
Solyc12g070200	Unknown Protein	1,88	1,53	-1,32
Solyc12g019680	Cytochrome P450	1,65	1,00	-2,00
Solyc12g044180	Disease resistance protein	1,61	1,11	-1,32
Solyc12g070160	Transposon Ty1-BR Gag-Pol polyprotein	1,46	1,18	-2,00

Selected candidate genes tested by qPCR are highlight in blue.

Supplementary Table 2. Primers used in this study.

Purpose	Gene	Name	Sequence (5' to 3')
qRT-PCR candidate genes	*Solyc12g042500*	Gibberellin-gene-F	GCTTTTGGTGGCACTTGTTC
		Gibberellin-gene-F	CCTGCCTTTGAACACCTAAAG
	Solyc12g044330	Aquaporin-F	GCCTTGCATAGCTTTTGGAC
		Aquaporin-R	AGCAATAGCTGAACCAACTCC
	Solyc12g049400	Jasmonate-ZIM-domain-gene-F	CAAAGCCTCAGCAACAAAGG
		Jasmonate-ZIM-domain-gene-R	GCCGCTGGCTTTATTTATTG
	Solyc12g038510	MADS-box-11-F (*J-2*)	TAGCCGCGGAAAACTCTATG
		MADS-box-11-R (*J-2*)	GTCCAAAGATGCGTAACTGC
	Solyc12g019320	Multidrug-resistance-gene-F	ATGTGGTGATGCTTGGTCTG
		Multidrug-resistance-gene-R	TCATAACCCCAGATGCAAGAG
	Solyc12g040800	C2 domain-containing-gene-F	CCTTGCTGTTCGTGATGTACG
		C2 domain-containing-gene-R	GAGAGGGTCAAATCTTCATTCC
	Solyc12g038520	Squamosa-promoter-gene-F	TGTTTCCCGTTCTCTGTTCC
		Squamosa-promoter-gene-R	AGAGAGCACAGCCAGAGTTTG
	Solyc12g044610	Myb-gene-F	TATTGAGCCTTCACCACTCTCC
		Myb-gene-R	CTCTGAGCTTTTGACTGGTGTG
Sequencing CDS	*Solyc12g038510*	MADS-11-CDS-F	ATGGGAAGAGGAAGAGTAGAACT
		MADS-11-CDS-F	TTAGAGCATCCACCCTGGAA
	Solyc12g042500	Gibberellin-CDS-F	ATGAAGCTAAAGTTTTCAACTTTG
		Gibberellin-CDS-R	TTAAGGACATTTAGGCCCTCCC
Genotyping *j-2* mutants	*Solyc12g038510*	PCR1-5'UTR-F	AACAGTGCTTCTCTCTCTTTCCA
		PCR1-Intron1-R	GGGCCCACATATAAGATTTCA
		PCR2-Intron1-F	GAGCATTGAACGTCTAACCTCA
		PCR2-Intron2-R	AAAAAGAATCACGGGCACAC
		PCR3-Intron2-F	TGAGTGGAAGTAAGGTTCGATCT
		PCR3-Intron4-R	AAAATGACTATCGAGGCATGG
		PCR4-Intron4-F	TTTTTAACTGGCCGCAAAGT
		PCR4-Intron7-R	TGTCAATATTTAATGCCGCTGA
		PCR5-Intron7-F	TGGTTTGGTGAAAAATAAAGAGAA
		PCR5-3'UTR-R	GGCCGGCAGTTGTTATTTT
qRT-PCR on pedicel (WT vs *j-2*)	*Solyc11g010570*	J-F	AAGGCAAATGAGGGGAGAAG
		J-R	CGGCTCAATCCAGTTTCAAG
	Solyc05g056620	MC-F	CCTTCAAAGGAACCACAAGC
		MC-R	GCTGTTGTTCCAAGTTTTGC
	Solyc03g114840	MADS1-F	TGCTACTTTGGAAGCCAACC
		MADS1-R	TCTCTGAGATCGTTGGAGGAG
	Solyc02g083950	WUS-F	TGGAGAAGAGCTTTAGGGAGTG
		WUS-R	GATCAACACCAACCCATGTC
	Solyc11g069030	Bl-F	AAAGAGGTCCATGGTCTCCAG
		Bl-R	CCTGCTTTTTGAGGAAGTGC
	Solyc07g062840	GOB-F	TGAACCTTGGGAACTTCCTG
		GOB-R	GTTGGATACTTCCGATCACG
	Solyc07g066250	LS-F	CGACATTAATCACGGGGTTC
		LS-R	TACCGGTGATTCGAAGAGTG
Housekeeping gene	*Solyc03g115810*	VAC-F	CGGAACGGATAATTCTGAGG
		VAC-R	CAGAATTGTTCCTCCACACG
in situ hybridation probes	*Solyc12g038510*	J-2-AntiSens-F	ATGGGAAGAGGAAGAGTAGAACT
		T7-J-2-AntiSens-R	TGTAATACGACTCACTATAGGGCTTAGAGCATCCACCCTGGAA
		T7-J-2-sens-F	TGTAATACGACTCACTATAGGGCATGGGAAGAGGAAGAGTAGAAC
		J-2-Sens-R	TTAGAGCATCCACCCTGGAA
qRT-PCR for MeDIP-PCR experiments	*Solyc12g038510*	MeDIP-PCR1-F	AAGACAGCTCGTAAGGGAAGC
		MeDIP-PCR1-R	TTTGGACACGAACATCAACG
		MeDIP-PCR2-F	GGGAGGCAGATGTGAGTTATGAGG
		MeDIP-PCR2-R	GCCTACTAAGTACACAAATTTG
		MeDIP-PCR3-F	CATTTATATGTGACTTATCAATTTG
		MeDIP-PCR3-R	TGGAAAGAGAGAGAAGCACTG
		MeDIP-PCR4-F	GTGTTTTTCTTGTTAGAGAC
		MeDIP-PCR4-R	CATTTCTTCTCTTAGCAAAAG
		MeDIP-PCR5-F	GTTGCTCTCATCATCTTCTCTAGC
		MeDIP-PCR5-R	CCGTCCTTTCTGTTTGTAGC
		MeDIP-PCR6-F	CCTCTCGGTTAATCGGTGTTCTTGG
		MeDIP-PCR6-R	GCCCGCATCTTGATCTGCCATAAAC
		MeDIP-PCR7-F	GGTGAACCACGTAAATCTGTTG
		MeDIP-PCR7-R	ACACATACGCACACACAAAAAG
		MeDIP-PCR8-F	TGAAATCTTATATGTGGGCCC
		MeDIP-PCR8-R	GTTGATAAACCTAATGGATG
		MeDIP-PCR9-F	GGGTTTTCCATGTTACCAATTC
		MeDIP-PCR9-R	CCTGCATAAGCAGCCTTTTC
		MeDIP-PCR10-F	GCAGCATGTTGGGAAAATTAC
		MeDIP-PCR10-R	GTCCAAAGATGCGTAACTGC
		MeDIP-PCR11-F	GCTCCTTCAACGTTCTCAAAGA
		MeDIP-PCR11-R	AATTGGTGCTCAAGCTGCTC
	Solyc12g038520	MeDIP-PCR12-F	TGTTTCCCGTTCTCTGTTCC
		MeDIP-PCR12-R	AGAGAGCACAGCCAGAGTTTG
Cloning Transposon on pGEMT-Easy	*Rider-J-2*	PCR1-5'UTR-F	AACAGTGCTTCTCTCTCTTTCCA
		PCR1-Intron1-R	GGGCCCACATATAAGATTTCA
Sequencing of pGEMT-Easy clones	*Rider-J-2*	pEasy-Rider-1	AACAGTGCTTCTCTCTCTTTCCA
		pEasy-Rider-2	GGGCCCACATATAAGATTTCA
		pEasy-Rider-3	CGGCTAGAGAAGATGATGAGAGCAAC
		pEasy-Rider-4	ATGGAGTCCGTAGACATTCAGATC
		pEasy-Rider-5	ATGAAGAGACTGCTGCTGGTCTG
		pEasy-Rider-6	GAGTGTGCTCATCCGCAACTAGG
		pEasy-Rider-7	CCCGCAGGGCAATTAACAGCTTC
		pEasy-Rider-8	CAAGGCATTCAACAAATCTCCACC
		pEasy-Rider-9	AAGATATTGCCCTAGTTGCGGATG
		pEasy-Rider-10	CCAGTGCCGTAGGAAGTTTGATGTATG
CRISPR-Cas9 edition on *J-2* gene	*Solyc12g038520*	sgRNA1	TGTGGTCTCAATTAGCTCCTTCAACGTTCTCAAGTTTTAGAGCTAGAAATAGCAAG
		sgRNA2	TGTGGTCTCAATTACATATTCTTGGAGAGGATTGTTTTAGAGCTAGAAATAGCAAG
Genotyping *CR-Slj-2* lines		CR-Slj-2 F	ATATTGAATCGTGTGATTGTCTC
		CR-Slj-2 R	TAACTTTCTTCAAAGATGCATCC
